# Effects of Using Perineal Underwear on Discomfort and Shame in Angiography Patients

**DOI:** 10.3390/ijerph18052480

**Published:** 2021-03-03

**Authors:** Eunhye Shin, Hanna Lee

**Affiliations:** 1 Neuro-Surgical Ward, Kyung Hee University Hospital, Seoul 02447, Korea; wnsl0501@naver.com; 2 Department of Nursing, Gangneung-Wonju National University, Wonju-si 26403, Korea

**Keywords:** coronary angiography, non-randomized controlled trials, perineum

## Abstract

The purpose of this study is to develop and apply a type of perineal underwear that protects the patient’s physical privacy and to examine its effects on perineal discomfort and shame. This study collected primary data from 44 patients who visited Kyung Hee University hospital in Seoul city and were admitted to the neurosurgery ward to undergo angiography between 7 August 2017, and 30 April 2018. In this quasi-experimental study with a nonequivalent control group posttest-only design, participants were divided into an experimental group (*n* = 22) and a control group (*n* = 22). The control group used conventional protection, which involved wearing padding around the perineum, while the experimental group wore the perineal underwear developed in this study. The underwear group showed a significantly lower degree of shame (Z = −5.39, *p* < 0.001) and perineal discomfort (Z = −5.88, *p* < 0.001) than the padding group. In the padding group, women felt significantly more shame than men did (Z = −2.48, *p* = 0.013). The use of the perineal underwear developed in this study significantly reduced the degree of shame and perineal discomfort in patients undergoing angiography. Such perineal underwear could also be useful for protecting patients’ privacy during perineal examinations.

## 1. Introduction

Privacy in the field of medicine can be explained in three dimensions: informational, physical, and psychological [1]. Informational privacy is defined as a patient’s perception of the regulation of personal information when a physician collects, uses, transmits, and stores information; physical privacy refers to the patient’s perception of the degree of their physical inaccessibility to others; and psychological privacy refers to the patient’s perception of the extent to which they are allowed by the physician to participate in decision-making and the degree of maintaining personal and cultural values such as inner thoughts, emotions, cultural beliefs, and religious customs.

In the past, patients have been placed in the weaker position as healthcare recipients without expert knowledge about medical and patient rights compared with healthcare providers, with little control over their personal privacy including their personal information, medical records, and physical exposure [2,3]. However, as the concept of privacy came to be understood as a fundamental human right to actively manage and control one’s own information and patients began to have an increased awareness of their rights, the need for the protection of patients’ privacy has increased [2,4]. Moreover, privacy is highly important in medical service as it is one of the most critical components of the quality of medical service and patient safety [1]. Violation of privacy may harm patients and impair the quality of healthcare [5], whereas protection of privacy increases their trust in the healthcare worker and motivates them to voluntarily provide personal information to medical institutions, thereby boosting the accuracy of medical information [6]. Protection of privacy not only enhances patient treatment but also has a positive impact on society by increasing the efficiency of the healthcare system [1]. Thus, patient management in hospitals must involve both therapeutic management and protection of personal privacy so as to maintain societal safety.

In this context, the global society has devoted a multilateral effort to minimize the potential violation of patients’ privacy and to secure the safety of medical information, such as by enacting information protection guidelines and laws [5]. In Korea, both the government and health organizations have been striving to protect patients’ privacy by including protection of physical privacy and care-related information, such as personal information, as one of the evaluation items for the accreditation of healthcare institutions [7].

Despite such effort to protect patients’ privacy, violation of patients’ privacy is highly likely to occur in the healthcare environment because of the disclosure of patients’ healthcare information and physical examination [6]. Particularly, violation of physical privacy is extremely likely during examination and testing. Studies on women [8,9,10] reported that women perceive having to expose their bodies for testing as an “essential but unpleasant experience.” Further, it has been reported that women feel that they have lost control of a situation as their body is partially exposed. A study on inpatients also confirmed that privacy is a universal psychological environment desired by all patients [11,12,13]. A violation of privacy has been reported to cause stress [12], loss of control, and shame [8,9,10] in patients. “Shame” refers to a painful feeling of lack of respect by others, and encompasses feelings such as embarrassment, humiliation, disgrace, and dishonor [10,11]. Additionally, responses to the violation of privacy differ between sexes as female inpatients are reported to be subject to a greater level of stress than their male counterparts [11,12,13,14].

Cerebral angiography is a procedure in which a catheter is inserted through the femoral artery to inject a contrast agent and X-rays are performed to observe abnormal structures, obstructions, or stenosis in the cerebral blood vessels for treatment [15]. For cerebrovascular angiography, the groin area, through which the catheter is inserted, is shaved to prevent infection on the day before the procedure. On the day of the procedure, the patient takes off his or her underwear and the perineum is then padded. The patient may also be requested to take off their underwear after the procedure for closer observation, which places them in a situation that may undermine their dignity. In this circumstance, any neglect in the effort to protect the patient from physical exposure would violate the patient’s privacy [16]. Further, as angiographies are performed on both sexes, it is possible to perform sex-specific comparisons of privacy.

Past studies on privacy have generally been focused on informational privacy [17,18], and the small number of studies on physical privacy have mostly been performed only on women [8,9,10,11]. Therefore, this study aimed to (1) develop and apply a perineal underwear for angiography to protect the patient’s physical privacy, (2) examine its effects on perineal discomfort and shame, and (3) explore the differences according to gender in patients admitted to a neurosurgery ward.

## 2. Methods

### 2.1. Research Design

This was a quasi-experimental research with a nonequivalent control group posttest-only design.

### 2.2. Participants

Forty-four patients who visited hospital Kyung Hee University hospital in Seoul city and were admitted to the neurosurgery ward to undergo cerebral angiography between 7 August 2017, and 30 April 2018, were enrolled in the study. The patients were divided into an experimental group (*n* = 22) and a control group (*n* = 22). The inclusion criteria were as follows: patients who provided informed consent to participate, were capable of understanding the questionnaire and had clear consciousness, were able to communicate, and were aged 18 years and above. Vulnerable research participants (patients with a visual, hearing, linguistic, or mental disorder); severely ill patients; emergency patients; and patients contraindicated for use of contrast media (pregnant women) were excluded from the study.

### 2.3. Instruments

#### 2.3.1. The Perineal Underwear

**Figure 1:**
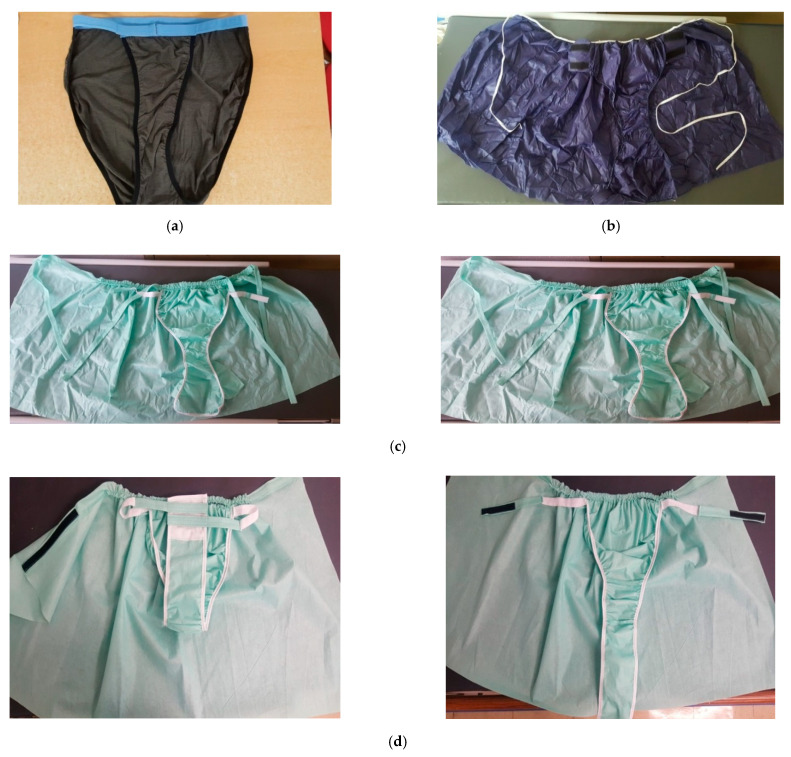
Developed Perineal Underwear. (**a**) First Version of the Perineal Underwear; (**b**) Second Version of the Perineal Underwear; (**c**) Third Version of the Perineal Underwear; and (**d**) Final Version of the Perineal Underwear.

The perineal underwear for angiography was developed and modified over a two-month period from 1 April to 31 May 2017. The proposed plan for developing the perineal underwear for angiography was validated by two neurosurgery professors, one neurosurgery head nurse, five angiography nurses, seven nurses with a minimum of five years of experience in the neurosurgery ward, and an expert team comprising nursing assistants. The development process of perineal underwear is shown in Figure 1.

The perineal underwear for angiography was first developed as a T-shaped type of cotton underwear (Figure 1a), but in light of increased cost, poor sanitation due to dust, and absorption of disinfectants such as Potadine Solution, the material of the underwear was changed to a nonwoven fabric. Considering infection control and practicality, the second model was developed using a disposable nonwoven fabric (Figure 1b). A lap skirt was then attached to the underwear such that the lap skirt would function as a blanket to cover the patient while waiting for the procedure. In the third development stage, the front part of the underwear that covers the perineal area was lengthened to run above the navel (Figure 1c). However, the perineal part of the underwear was too wide and thus was at risk of Potadine absorption; thus, the corresponding part was made to be narrower. The length of the lap skirt was adjusted from below the knee to the knee level. Since the sides of the perineal underwear were too tight and did not fit well during trials, double-sided tape was placed on the sides. A sticker was then added to the underwear to prevent it from moving during the procedure. In the fourth development stage, the underwear remained at risk of Potadine absorption even after applying double-sided tape on the sides; thus, after a discussion among the angiography team, silk plaster was applied to the outside part. To make changing out of underwear easier in case the patient has to use the bathroom in the angiography room, the waistband was changed from a string fixation clip to a Velcro fastener. Finally, various sizes of the underwear were produced, and the shape, texture, size, and line of the underwear as well as the skirt waistband and length were checked in order to come up with the final product (Figure 1d).

#### 2.3.2. Measurement

The patients were instructed to rate perineal discomfort using a 10-point graphic rating scale throughout the examination process. A higher score indicated a higher degree of perineal discomfort. The patients were also instructed to express and rate their shame using another 10-point graphic rating scale throughout the examination process. A higher score indicated a higher degree of shame.

### 2.4. Study Procedure

In order to verify the effectiveness of the perineal underwear, we collected data at Kyung Hee University hospital in Korea. Data were collected at different time points for the experimental group and control group to prevent diffusion of treatment. Data for the padding group were collected from August to November 2017, while those for the underwear group were collected from November 2017 to April 2018. Both groups underwent shaving of the perineal area the day before angiography and were informed about the precautions related to the procedure. On the day of angiography, the control group was provided with padding for use over the perineal area, which is the conventional approach, and the experimental group was provided with the developed perineal underwear. The intervention was administered by a male healthcare provider for male patients and a female healthcare provider for female patients. The aforementioned survey about perineal discomfort and shame was then administered at the ward, and vital signs were assessed 15 min after the patients returned from angiography. The principal investigator collected the data after explaining the study to the patients and obtaining written informed consent forms from them. Equal amount of knowledge was shared with both the experimental and control groups in relation to angiography with the help of the materials and guidelines utilized in the ward.

### 2.5. Data Analysis

The scores for perineal discomfort and shame were analyzed with the Mann–Whitney U test using SPSS Statistics for Windows, version 23.0.

### 2.6. Ethics Approval and Consent to Participate

This study was approved by the Kyung Hee University Hospital Institutional Review Board (approval no. 2017-06-066) and was conducted on patients who were admitted for angiography. A consent form was attached to the questionnaire, and the signed forms were collected after the participants received a verbal explanation about the study.

## 3. Results

### 3.1. Effects of Wearing Perineal Underwear

After angiography, the scores for shame and perineal discomfort were compared between the padding group and underwear group using the Mann–Whitney U test. The underwear group (M = 1.23) showed a significantly lower degree of shame than the padding group (M = 6.59) (Z = −5.39, *p* < 0.001). The underwear group (M = 7.77) also showed significantly less perineal discomfort than the padding group (M = 0.23) (Z = −5.88, *p* < 0.001) (Table 1).

### 3.2. Effects of Wearing Perineal Underwear According to Gender

The degree of shame and perineal discomfort were also compared between the two groups according to sex. In the padding group, women (M = 7.58) showed a significantly greater degree of shame than men (M = 5.40) (Z = −2.48, *p* = 0.013), while there were no significant differences in perineal discomfort between men (M = 7.30) and women (M = 8.17) (Z = −1.16, *p* = 0.245). In the underwear group, there were no significant differences in the degree of shame between men (M = 0.83) and women (M = 1.70) (Z = −0.99, *p* = 0.323). There were also no differences in perineal discomfort between men (M = 0.25) and women (M = 0.20) (Z = −0.27, *p* = 0.785) (Table 2).

## 4. Discussion

This study developed a type of perineal underwear for angiography to protect the physical privacy of patients and verified its effects on perineal discomfort and shame. The following results were obtained.

First, the use of the perineal underwear significantly reduced the patients’ degree of shame. This finding may be due to the fact that the underwear we developed can be worn by patients and has a lap skirt to cover their legs even after removing their blankets, which minimizes physical exposure and subsequent shame. According to a previous study on patients’ privacy, more than 91% of patients are concerned about their physical privacy [16], suggesting the importance of protecting physical privacy to reduce anxiety, increase patients’ trust in healthcare professionals, and boost therapeutic outcomes. Thus, the perineal underwear used in this study could be applicable in neurosurgery, cardiology, and neurology during tests that involve the femoral artery, such as angiography, neurovascular stenting, and Guglielmi detachable coiling. It could also be used in other perineal examinations such as those in the field of obstetrics and gynecology. According to a previous study conducted in the emergency department [6], establishing an ethical environment can significantly improve patients’ privacy and satisfaction; thus, efforts to protect patients’ privacy would lead to increased patient satisfaction.

Second, the use of the perineal underwear significantly reduced the patients’ perineal discomfort. Compared with the conventional method, the use of the underwear involved applying plaster firmly on the perineum, including the area above the pubic hair and the area near the anus, and the underwear needed to be fixed only to the sides of the perineal area. This technique seemed to have contributed to reducing the discomfort in the area that would have been caused by removing the padding.

Third, we found a significant difference in the degree of shame according to sex in the padding group. Although there are very few studies with which we could compare this finding, it is similar to that reported by a study that investigated care experiences in the hospital according to sex [19], where female patients wanted more privacy as well as pain control than male patients. This finding suggests that women are more sensitive to exposing their perineal area than men, which may be related to social and cultural factors. A Taiwanese study on female adolescents [8] reported that cultural factors such as concerns for perineal exposure and confidentiality hinder their utilization of obstetrics and gynecology care. Additionally, a Korean study on women undergoing genital testing [10] observed that having to remove items of clothing and underwear strip women of their perceived dignity and nobility. A study on Swedish women undergoing pelvic examinations revealed that friendly guidance and explanations given by physicians and examiners and a trusting relationship with the examiner converted patients’ negative feelings to positive experiences and helped them accept the situation [9]. On the other hand, Korean women were found to avoid or postpone visiting the hospital or only passively accept the situation [10], suggesting that cultural and social differences may be at play regarding perineal exposure. It is speculated that women in East Asian cultures tend to think of perineal exposure as an embarrassing situation and thus want to avoid it owing to the stronger conservative views of sexuality in these cultures [11]. Further, previous studies reported that patients tend to prefer physicians of the same sex, and this preference is more evident when it comes to genital testing [20,21]. As perineal preparation for testing is performed by health care providers of the same sex in our hospital, further studies are needed to examine whether feelings of shame would differ if healthcare providers of the opposite sex were involved. This further suggests that women would be more satisfied with wearing the developed perineal underwear and warrants further studies on the perception of privacy according to sex.

As most of the previous studies have focused on protecting medical informational privacy [17,18], with few studies examining physical privacy, our study is significant because it developed a type of perineal underwear that is feasible for commercialization which can contribute to the protection of patients’ physical privacy.

## 5. Conclusions

This study developed a type of perineal underwear for the protection of the physical privacy of patients undergoing angiography. The results showed that the use of such perineal underwear significantly reduced the degree of shame and perineal discomfort among the patients. Such perineal underwear could also be useful in protecting the privacy of patients during other procedures involving perineal examination. Since this study was conducted on a small sample of patients who underwent angiography in one university hospital, our findings have limited generalizability. Further studies are required to investigate the use of such perineal underwear in a larger sample. Further, we only assessed the effects based on shame and perineal discomfort in our study, so subsequent studies should also explore the effects using other validated instruments.

## Figures and Tables

**Table 1:** Effect of wearing perineal underwear.

	Control Group (*n* = 22) (M ± SD)	Experimental Group (*n* = 22) (M ± SD)	Z	*p*
Shame	6.59 ± 2.04	1.23 ± 1.72	−5.39	<0.001
Perineal discomfort	7.77 ± 1.54	0.23 ± 0.43	−5.88	<0.001

**Table 2:** Effects of wearing perineal underwear by gender.

		Male (*n* = 10) (M ± SD)	Female (*n* = 12) (M ± SD)	Z	*p*
Control Group	Shame	5.40 ± 1.71	7.58 ± 1.78	−2.48	0.013
	Perineal discomfort	7.30 ± 1.49	8.17 ± 1.53	−1.16	0.245
Experimental Group	Shame	0.83 ± 1.40	1.70 ± 2.00	−0.99	0.323
	Perineal discomfort	0.25 ± 0.45	0.20 ± 0.42	−0.27	0.785
